# Supporting linkage facilitators working with persons with opioid use disorder: challenges, advances, and future directions

**DOI:** 10.3389/fpubh.2025.1587101

**Published:** 2025-09-01

**Authors:** Tess K. Drazdowski, Anthony P. Coetzer-Liversage, L. A. R. Stein, Martha Tilson, Dennis P. Watson, Lauren A. Hoffman, Aaron Hogue, Sierra Castedo de Martell, Angela Marie Hagaman, R. Neil Greene, Milan F. Satcher, Patrick F. Hibbard, Jodie M. Dewey, Ashli J. Sheidow, Noel Vest

**Affiliations:** ^1^Chestnut Health Systems, Bloomington, IL, United States; ^2^ServeMinnesota, Minneapolis, MN, United States; ^3^Department of Psychology, University of Rhode Island, Kingston, RI, United States; ^4^Department of Behavioral and Social Sciences, School of Public Health, Brown University, Providence, RI, United States; ^5^Rhode Island Department of Behavioral Healthcare, Developmental Disabilities and Hospitals, Cranston, RI, United States; ^6^Center on Drugs and Addiction Research, University of Kentucky, Lexington, KY, United States; ^7^Recovery Research Institute, Center of Addiction Medicine, Massachusetts General Hospital and Harvard Medical School, Boston, MA, United States; ^8^Partnership to End Addiction, New York, NY, United States; ^9^The Phoenix, Boston, MA, United States; ^10^East Tennessee State University, Addiction Science Center, College of Public Health, Johnson City, TN, United States; ^11^University of New Mexico, Albuquerque, NM, United States; ^12^Department of Community & Family Medicine, Dartmouth Hitchcock Medical Center, Lebanon, NH, United States; ^13^The Dartmouth Institute of Health Policy, Giesel School of Medicine, Dartmouth College, Hanover, NH, United States; ^14^The Dartmouth Institute of Health Policy & Clinical Practice, Giesel School of Medicine, Dartmouth College, Lebanon, PA, United States; ^15^Department of Community Health Sciences, School of Public Health, Boston University, Boston, MA, United States

**Keywords:** linkage facilitation, opioid use disorder, medication for opioid use disorder, workforce support, professional standards

## Abstract

This narrative review explores the evolving role of linkage facilitation (LF) in supporting persons with opioid use disorder (OUD) including both the organizational strategies to initiate and sustain LF services and strategies to support the LF workforce. Drawing on expert consensus and iterative review by an interdisciplinary author team, we synthesized relevant literature from diverse fields using a narrative review approach. Organizational strategies include: ensure leadership support, engage community partners and tailor services, consult with those already delivering LF services, provide adequate pay and career advancement opportunities, establish role clarification, and create official documentation of LF services. Strategies to support the LF workforce include: ensure comprehensive training and continuing education, provide robust supervision, encourage self-care, and establish quality/fidelity standards. Recommendations for advancing the profession include enhancing training for both LFs and supervisors, establishing centralized resource libraries, and tailoring support for diverse OUD-affected populations. This review advocates for the development of best practice guidelines, practical evaluation tools, and a collaborative resource-sharing hub to ensure long-term LF workforce sustainability and improved outcomes for those served.

## 1 Introduction

This narrative review examines the research and practice literature pertaining to supporting people delivering linkage facilitation (LF) for persons with opioid use disorder (OUD). Opioid misuse remains a national healthcare problem, with alarming rates of OUD and lethal opioid overdoses across demographic groups (1, 2). LF—also called service linkage or linkage to care—encompasses a range of services to help people access appropriate treatment, harm reduction, and support interventions, maintain medication and treatment adherence, and obtain resources that assist immediate and long-term recovery goals (3). LF may be especially helpful to persons looking to start medications for the treatment of OUD (MOUD) given the considerable barriers to treatment access and initiation (4). LF for MOUD service uptake is employed in various settings including primary care, emergency departments, behavioral health, perinatal care, the criminal legal system, and harm reduction facilities (3). Recent empirical reviews suggest LF can foster MOUD initiation and adherence, promoting access to and engagement in other supportive behavioral and social services (5–7).

**Figure 1:**
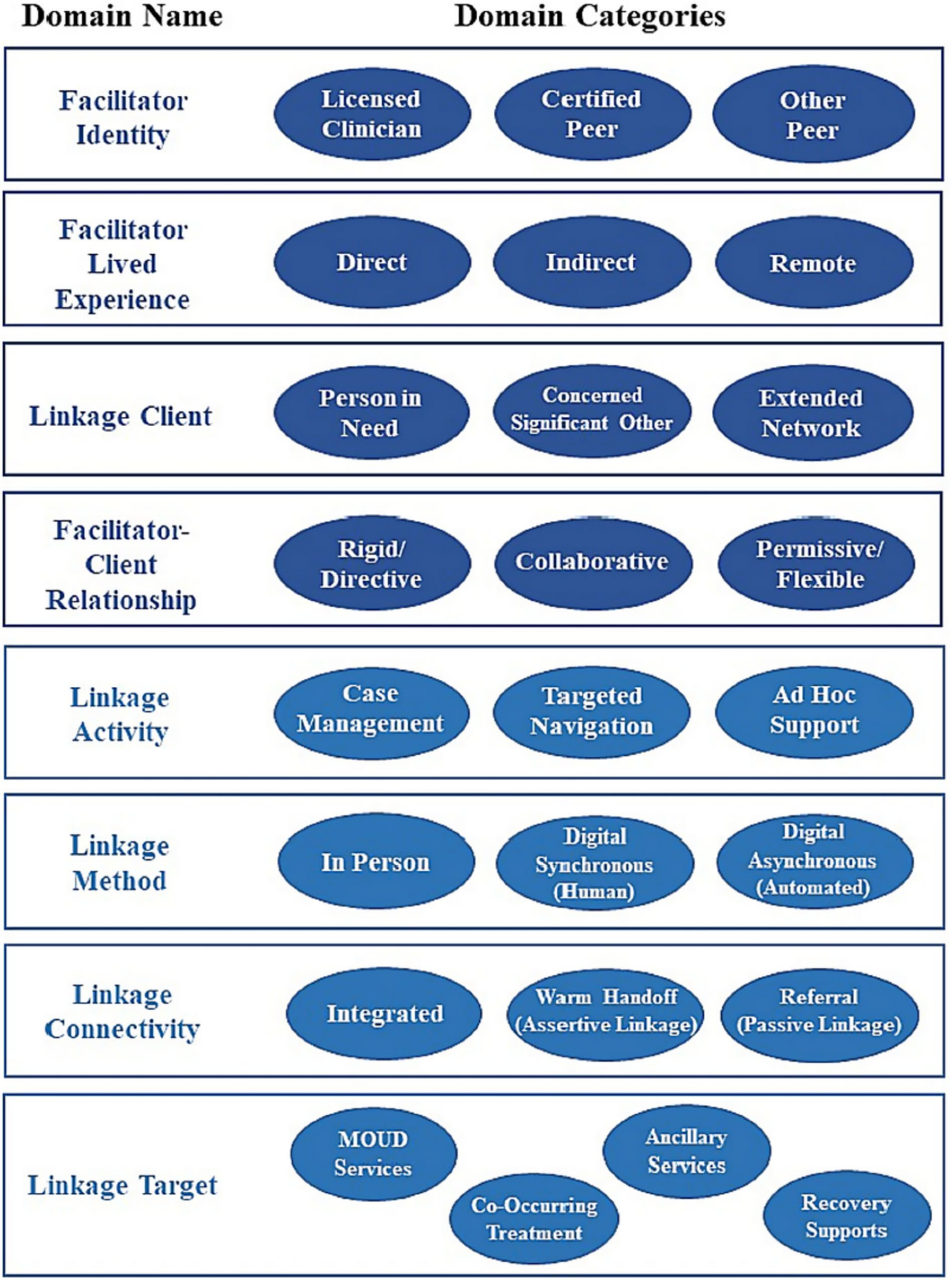
Taxonomy of linkage facilitation for OUD services from Hogue et al. (8).

LF can be delivered by an array of practitioners, aims to achieve a host of linkage goals, and consists of multifaceted linkage activities. To promote consistent communication about standards and practices, Hogue et al. (8) describe a taxonomy of LF services for persons with OUD that contains eight dimensions (see Figure 1): *facilitator identity*; *facilitator lived experience*; *linkage client*; *facilitator-client relationship*; *linkage activity*; *linkage method*; *linkage connectivity*; and *linkage goal*.

LF for people with OUD is becoming increasingly commonplace (3), and the LF workforce is becoming larger and more diverse. As such, linkage facilitators represent various professions including social workers, nurses, case managers, health navigators, community health workers (CHW), and peer recovery support specialists (PRSS). Therefore, workforce retention must consider such factors as facilitator professional identity, potential experience with substance misuse and recovery, and contextual factors within the workplace (9).

Linkage facilitators face multiple challenges in deploying their services, including others’ stigma toward clients (and, at times, toward facilitators with lived experience of recovery), inadequate supervision, role ambiguity, and a lack of integration within healthcare teams (10–12). These issues, compounded by the scarcity of resources to connect individuals with OUD to appropriate services, often lead to burnout and turnover, particularly among professionals like health navigators, CHWs, and PRSS (13–15). Additionally, poor integration into healthcare teams and being assigned duties beyond their scope further contribute to role dissatisfaction (16–20). Linkage facilitators also face financial instability due to inadequate compensation and limited career advancement opportunities, which can create financial fragility and increase turnover rates (19, 21). Combined with stressors such as vicarious trauma and unsupportive work environments, these factors heighten vulnerability to stress and burnout (19, 22). Addressing these challenges is essential to ensuring this vital workforce is supported and retained. Building upon the critical need for supporting linkage facilitators outlined in the introduction, this paper now details the systematic approach taken to review the existing literature and identify key strategies for their sustained effectiveness.

## 2 Methods

We conducted this review as a collaboration among researchers associated with two large initiatives supported by the National Institute on Drug Abuse, the Consortium on Addiction Recovery Science (CoARS) and the Justice Community Opioid Innovation Network (JCOIN). We determined a narrative review as the most appropriate approach because of the nascent state of empirical literature spread across various disciplines that is directly related to LF practice for persons with OUD that would be necessary to conduct a systematic review (23–26). Although narrative reviews are sometimes critiqued for lacking rigor, it is important to recognize their strength in providing extensive coverage of literature and flexibility to emerging knowledge and concepts (27). A key outcome of narrative reviews is a more in-depth understanding of the topic of focus (28).

All review authors are highly engaged practitioners and researchers in the area of LF services and this review drew primarily on their knowledge of the disparate LF literature. We met approximately monthly via Zoom and conversed through frequent email correspondence over 12 months to prioritize the review goals, identify critical themes in the field, assign specific subtopic writing responsibilities, and review and co-edit through an iterative revision process. After all authors agreed on an outline through consensus and selected subtopics to complete initial drafts based on interest and expertise, authors identified articles through online searches in research literature databases and using their expertise and knowledge related to specific content areas within the review’s aim. In drafting the review, we were guided by Ferrari (29) to select articles that include linkage facilitation services as described in Hogue et al. (8). Authors within subtopics worked together to select appropriate references. After the initial draft all authors reviewed the paper in its entirety, suggesting additional references as appropriate. While all authors revised and approved the final manuscript, the first and second author were responsible for the final review of the text and selection of the literature supporting the key recommendations. While narrative reviews are subjective, as is all research, they add meaningful contributions to the literature (30). Having established the methodology employed in this narrative review, we now present the findings, categorizing them into key strategies for organizational support and workforce development that emerged from the literature.

## 3 Results

We first discuss approaches for supporting linkage facilitators working with persons with OUD and end with how the field can advance quickly to support this rapidly growing workforce. Due to the current state of the literature on LF to MOUD and our expertise in this area, many of our examples are framed from the perspective of professionals, such as PRSS and CHWs. To better understand how the field can support and grow the LF workforce, we first examine organizational-level strategies that facilitate the successful initiation, implementation, and sustainment of LF services for individuals with OUD.

### 3.1 Organizational strategies to initiate and sustain LF services

Organizational strategies to support initiation and sustainment of LF services for those working with persons with OUD include leadership engagement, flexible work environments (31), learning collaboratives, external facilitation, didactic webinars (32, 33), and full-time employment (34). Similarly, enablers of adoption and delivery include government-supported programs that create LF positions within organizations (31), strategic plans for employing linkage facilitators (35), and Executive Orders assigning linkage facilitators to primary care sites (36). Work with PRSS linkage facilitators has shown that these workers may not become integral to services if stakeholders are unwilling to integrate them into existing practice (11). A systematic review suggests incorporating linkage facilitators into systems is enhanced by linkage facilitators having a peer network and organizational resources (e.g., internet access), preparing staff through training (e.g., how to interact with linkage facilitators) and role clarification, and attending to staff attitudes towards linkage facilitators (11). This is particularly important given that lack of role clarity has been found to lead to feelings of exclusion, tokenism, and stigmatization among PRSS linkage facilitators within another review (37). Implementing and maintaining LF services for working with persons with OUD requires comprehensive strategies that address organizational needs and overcome barriers.

#### 3.1.1 Ensure organizational leadership support

The literature indicates commitment of organizational leadership as vital to supporting linkage facilitator activities (11) and is especially germane when clarifying the need, importance, and practices of linkage facilitators and initiating policy changes to support them (38). Early evidence points toward transparent backing from organizational leadership as an essential element for fostering the integration of LF services by openly advocating for their adoption within the organization, and securing formal endorsement from leadership and their active involvement in decision-making processes as a critical factor for garnering organizational support (39, 40). For example, such support may be critical for creating pathways to employing LFs with lived experience and for reaching patients with disproportionately high risk of limited access to MOUD (e.g., during incarceration) and fatal overdose (e.g., upon reentry) (41). While lived experience enhances the impact of linkage facilitation, LFs with misdemeanor or felony histories may be unemployable without changing an organization’s hiring policies (124). Likewise, leader-to-leader advocacy may be needed to establish cross-system policies and agreements to allow PRSS or other LFs with lived experience to contact clients during incarceration or community supervision (125, 126).

#### 3.1.2 Engage community partners and tailor services

Once there is support from organizational leadership regarding LF service provision, involving key community partners (e.g., representatives from organizations to which linkage is being facilitated to, staff and team leaders, as well as consumers who have received or may benefit from LF services), in the design and implementation of those LF services fosters buy-in and encourages collaboration. Customizing LF services to align with requirements of the organization looking to deliver LF services, such as defining tasks, schedules, and workflows, is instrumental in achieving successful integration (42). As this can vary greatly from setting to setting, particularly given the range of treatment options and LF locations for those working with persons with OUD, involving community partners can be essential for successful LF program implementation and linkage facilitator integration within the organization and community. Further, engaging community partners can expand the available resource network for an organization, thereby reducing concerns associated with resources shortages with clients.

#### 3.1.3 Consult with those already delivering LF services for people with OUD

Organizations who want to provide LF services to people with OUD should decide if the LF services will be a stand-alone role or if the services will be added to an existing role, as well as whether LF services occur internally or in collaboration with an outside agency. Financial considerations, along with considering adding burden to systems that may already be operating at maximum capacity, as well as decisions of each domain of the LF taxonomy [see Figure 1 (8)], ought to guide organizations. To manage these multiple decision points, utilizing outside LF organizations or consultants (e.g., community and lived-experience advisory boards) can assist agencies in LF program development by collaborating on developing policies, training programs, and standards to support facilitator integration. These initiatives should focus on the strategies highlighted above including defining roles, procedures, boundaries, and resource allocation to ensure successful implementation (40, 43). Ideally, consultants will include individuals who have LF experience. Additionally, for programs looking to create LF programs to assist persons with OUD, hiring and working with consultants that have expertise working with that specific focus would be exemplary.

#### 3.1.4 Provide adequate pay and career advancement opportunities

While many linkage facilitators are generally satisfied with their work, common areas of dissatisfaction include low pay and limited opportunities for advancement (19, 44). Higher pay for such workers has been positively associated with job satisfaction, therefore it is imperative that organizational support includes adequate pay and benefits and opportunities for career advancement for linkage facilitators. Research on mental health peer workers suggests that these same factors contribute to workers leaving the field to pursue other careers (13) and similar dynamics may be present among SUD peer workers (45).

Medicaid billing and reimbursement processes can be overly complicated and pay for only selected services (46). Thus, the evidence suggests it is important for agencies that provide LF services to be proactive and intentional about fiscal sustainability for these cost-effective services (47) and the LF workforce. Linkage facilitators often work in positions that cannot financially support them long-term as one residential treatment administrator explains, “… we are asking these people to impact our communities for the better yet we are not giving them a wage that can support a life or raising a family” (48). Setting specific role clarification and ongoing documentation of LF outcomes can lead to a more equitable and sustainable career pathway for the LF workforce.

#### 3.1.5 Establish role clarification

Linkage facilitators often operate in environments where their roles and responsibilities may be poorly defined (37) which can result in a variety of challenges in providing services (49), such as other staff members not understanding the linkage facilitator role, questioning linkage facilitator credibility, and devaluing LF services (20). Additionally, a clash in philosophies between a medical model, where an expert knows best, versus experiential knowledge and client self-determination may preclude integration of some linkage facilitators within systems (38). The need for clarity in roles and responsibilities has been indicated as an essential factor influencing effective implementation into existing systems (40, 50). Preparing staff for the introduction of new LF roles can help with integration. Clarification can be accomplished in part via staff training so that linkage facilitators and other staff have a common understanding of purpose, roles, and activities of linkage facilitators (51, 52).

#### 3.1.6 Create official documentation of LF services

As LF services are being established, it is important to formalize the commitment to initiate and maintain LF services through written policies and procedures, which ensure clarity and accountability. Official documentation also helps to define and formalize facilitator roles that can guide them in their work. Developing comprehensive documents that outline decisions, policies, and procedures in collaboration with leadership facilitates transparency and consistency (40, 43), helping to support both the services being provided and the linkage facilitator workforce.

### 3.2 Strategies to support the linkage facilitation workforce

While establishing robust organizational strategies is fundamental for the successful implementation of LF services, equally crucial are direct approaches aimed at nurturing and sustaining the individual linkage facilitation workforce. In this section, we highlight several strategies that would be beneficial for supporting linkage facilitators to improve workforce retention and other outcomes. Many of the strategies presented are also approaches to supporting LF service implementation more broadly (53), as well-implemented programs should result in more supportive work environments for linkage facilitators.

#### 3.2.1 Ensure comprehensive training and continuing education

Training for linkage facilitators varies by organization and is often tailored to facilitator identity, lived experience, client needs, and available resources (8). Many linkage facilitators come from professions such as licensed clinicians, certified PRSS, or CHWs, each with state-specific training standards. Coordination with credentialing bodies may ensure consistency in basic knowledge and training requirements. Specific to working with persons with OUD, training should address MOUD, opioid withdrawal, harm reduction strategies, available community resources, and the intersecting risks and systems (e.g., criminal legal systems, child welfare systems) that impact care access, continuity, and outcomes. Linkage facilitators work within a client-centered framework, meaning they support clients without imposing change, using techniques like motivational interviewing to meet clients where they are in the behavior change process (54, 55). Training that includes specific treatment models or work settings, such as primary care, can further ensure linkage facilitators are prepared for success (56). Additionally, experienced linkage facilitators should have a key role in developing and revising training programs, as recommended in PRSS standards (57). It is important to recognize that in some settings, access to training and certification represents barriers to becoming a linkage facilitator and can further exacerbate health inequities that already exist at a systemic level (58). Scholarships (as noted below) may in part offset such barriers but will likely not eliminate them. Systems must be mindful of such inequities and how best to address them for a skilled, diverse, and representative workforce.

Ethical considerations are also vital in LF training. Most LF professions have established codes of ethics, such as NAADAC’s (Association for Addiction Professionals) guidelines, that can provide a foundation for those lacking ethics training in their specific field (59). Linkage facilitators should be able to identify fraud and abuse within referral organizations and recognize ethical dilemmas within their own organizations, such as being pressured to make internal referrals that may not benefit clients (60). Additionally, they should be aware of their own biases regarding recovery pathways, including MOUD and harm reduction (61). Younger linkage facilitators with lived experience may face unique challenges in setting boundaries, which can increase stress and require additional support (62, 63). Ethical training is essential for maintaining professional boundaries, avoiding burnout, and empowering clients rather than fostering dependency.

Training should also prepare LFs to navigate ethical and legal challenges specific to subpopulations with OUD. For example, pregnant and postpartum patients face elevated risks of medicalized stigma, criminalization, and loss of parental rights (127, 128). Some states even require automatic report of child abuse when prescribed MOUD is taken during pregnancy (129). LFs supporting this population need clear guidance on their jurisdiction’s reporting requirements—and a strong understanding of their limits—to ensure they protect patients’ privacy rights to the fullest extent allowed by law.

Additionally, continuing education (CE) is a crucial component of ongoing support for linkage facilitators and has been emphasized for over a decade within the PRSS workforce (64). CE requirements vary by state (65), but SAMHSA (57) recommends annual CE on ethical standards, with provisions for offsetting costs, such as scholarships. CE should also cover recent research in mental health, trauma, MOUD, and unique considerations for serving subpopulations while providing opportunities for mentoring, skill development, and peer learning (64). A program support team should oversee the development and delivery of CE (64). Additionally, several studies indicate that regular CE helps linkage facilitators integrate more effectively into healthcare settings, enhancing their legitimacy and reducing their experience of stigma and discrimination (38, 40, 66). Certification and ongoing professional development can clarify LF roles within teams, improving job satisfaction and workforce retention.

#### 3.2.2 Provide robust supervision

Ongoing supervision and debriefing are crucial for the successful implementation of linkage facilitators, particularly for those working with individuals with OUD (11, 67). However, literature suggests that LF supervisors may not provide adequate support, either due to insufficient supervision or lack of knowledge and skills (12, 52, 68). Despite these challenges, regular supervision remains essential to addressing issues like burnout and promoting job satisfaction and success among linkage facilitators (22, 31, 69). Adequate supervision may also promote linkage facilitator resiliency in the face of work-related stressors (22, 46). Young linkage facilitators working with youth may encounter difficulties, for example, in role clarification and boundaries; however, provision of supervision, particularly emotionally supportive supervision can enhance success (70, 71). Supervisors who are responsive, flexible, and encourage autonomy can reduce professional isolation, help establish role clarity and signal the value of linkage facilitators within their organizations, especially when adopting trauma-informed approaches that account for the lived experiences of linkage facilitators (15, 72). Feeling respected and supported by supervisors enhances job satisfaction (73), and regular, constructive feedback is recommended to sustain effective supervision, supported by organizational policy and financial resources (74). Various guidelines, including those from SAMHSA and the National Association of Peer Supporters (NAPS), provide frameworks for effective LF supervision (75–77). Supervisors should also model self-care and boundary-setting, support personal and professional growth, and have knowledge of local OUD services and harm reduction strategies (76, 78).

Supervision should also account for the varied types of lived experience among linkage facilitators, which range from direct, indirect, and remote lived experiences (8). Direct lived experience can positively impact professional roles but may also result in stigma, especially when disclosed (12, 79–83). Indirect and remote lived experiences present different challenges that require tailored supervisory support to help linkage facilitators navigate their roles and client relationships. Supervisors with direct lived experience can offer unique integration and role clarity benefits for linkage facilitators who share similar backgrounds (12, 84).

#### 3.2.3 Encourage self-care

As most linkage facilitators working with persons with OUD support high-acuity populations, many while maintaining their own recovery, their work can be challenging (85). Indeed, a unique aspect of LF is that workers can be chosen specifically for their lived experience, with potentially relapsing conditions, and yet ironically self-care (including taking time off) to manage conditions is recognized by some linkage facilitators as jeopardizing employment (12).

Research with PRSS linkage facilitators has identified that self-care routines are important to mitigating burnout and improving resiliency against chronic work stressors (86, 87), findings which likely have broad applicability to others in linkage facilitator roles. Self-care can be promoted through professional development opportunities (e.g., trainings) and funding structures (46), as well as organizational policies promoting flexible scheduling and mental/emotional support. Self-care is enhanced by a sense of community, self-awareness (e.g., of triggers), boundaries between work and home, and ongoing training and supervision (12, 88). For example, for LGBTQ+ linkage facilitators, self-care is supported when organizations build partnerships with affirming providers, ensure access to evidence-based, gender-affirming care, and recruit staff with relevant lived experience and cultural competence (89, 90). However, it should be noted that the assumption that linkage facilitators are particularly vulnerable to stress is largely untested (87). Finally, while employee-level self-care practices are essential, workplaces must avoid precluding them. Supervisors and organizations working with linkage facilitators should be educated on the occupational hazards of the linkage facilitator role (e.g., vicarious trauma, burnout) and adopt a trauma stewardship approach [i.e., policies and practices that prioritize the well-being, resilience, and sustainable self-care of employees in psychologically hazardous roles (91)].

Of note, particularly for linkage facilitators with direct lived experience, a potential point of conflict exists if the resources that a linkage facilitator uses to support their own recovery as part of their self-care routine overlaps with the resources that the client is using (92). This potential underexplored challenge may exist more in areas with limited resources, such as rural areas, and has not been sufficiently studied to date. Organizations supporting linkage facilitators should have ways to address this challenge, including comprehensive supervision as noted above and provide the infrastructure and support to access other forms of support, such as attending online support groups that could be located outside of the local recovery support network.

#### 3.2.4 Establish quality/fidelity standards

Establishment of quality standards for LF would help define processes and expected outcomes for the field that could help linkage facilitators better clarify their role within their organization and their identities as related to the LF profession. A good place to start establishing such standards is through the identification of LF fidelity guidelines. The most important dimensions of fidelity have been defined as *adherence* (the specific content or quantity of interventions delivered) and *competence* [the technical skill or quality of delivery (93)]. Fidelity includes knowledge of client issues, intervention appropriateness and timing, and degree of responsiveness to client behaviors (94, 95). Formalizing LF fidelity standards will require detailed descriptions of development activities beyond what is captured in the current LF literature [e.g., (96–98)]. More rigorous evaluation of LF fidelity tools and procedures in clinical settings is needed to support consistent, high-quality LF delivery that conforms to the procedures and goals of LF models over time. Three principles should govern LF fidelity advances. First, tools and procedures used to evaluate fidelity should reflect the multiple domains that constitute LF for OUD [(8); see Figure 1] and the core tasks of the specific LF model (99). For example, one of the authors (REDACTED) developed a fidelity tool for an intervention in which PRSS linkage facilitators collaboratively link adults with OUD to recovery support services. Because the facilitator-client relationship is deemed “Collaborative” by tool developers, the tool includes coding criteria regarding the facilitator’s consideration of patients’ desires/concerns, while supporting the goal of service linkage. Second, behavioral protocols should articulate procedures for training and monitoring practitioners in *how to* use companion fidelity tools. Well-established methods for successful practitioner training [e.g., behavioral rehearsal with active feedback, ongoing expert consultation (100)] should be repurposed for fidelity training to support the LF workforce. Third, LF fidelity methods need to be pragmatic, that is, practitioner-friendly, compatible and integrated with routine services, minimize missing data, and facilitate user-reactivity tracking (101). Note there are documented advantages and burdens associated with each of the primary fidelity evaluation methods: independent-observer or supervisor report, practitioner or client self-report, and case record review (102).

Creating quality fidelity guidelines can then support providing feedback to linkage facilitators and lead to continuous improvement in services. Performance feedback and evaluation play pivotal roles supporting linkage facilitators by improving implementation and maintenance of LF services. By setting up regular feedback mechanisms, organizations can effectively monitor progress, pinpoint challenges, and adapt as needed. Consistent evaluation of LF services, encompassing tasks such as monitoring daily activities and evaluating their effectiveness, is fundamental for ensuring their long-term sustainability (50).

## 4 Discussion: promising avenues for advancing the profession of linkage facilitation for OUD

Drawing from the comprehensive findings on effective strategies for supporting linkage facilitators, this discussion synthesizes these insights and outlines promising future directions for advancing the profession of linkage facilitation for opioid use disorder. The successful initiation and sustainability of LF services require a multifaceted approach that involves professional development and growth opportunities, external facilitation, leadership support, stakeholder engagement, feedback mechanisms, and documentation. This rapidly maturing field can build upon these components as it prepares the future LF workforce and the settings in which they are called upon to contribute their skills to support individuals with OUD. Below, we offer some recommendations for facilitating that progress in the coming years (see Table 1).

**Table 1:** Summary of key recommendations.

Recommendation	Brief description
1. Enhance trainings for LF	As LF gain recognition as a distinct role in OUD care, there is a pressing need for inclusive guidelines and training to support supervisors and coworkers without creating credentialing barriers
2. Create library for LF services	A centralized resource hub is needed to support organizations providing or launching LF services by offering tools, research, guidance, and networking to strengthen the workforce and improve service delivery
3. Improve understanding of tailored LF services	Expanding LF services requires tailored training and research to meet the unique needs of diverse populations with OUD, including pregnant people, LGBTQ+ individuals, youth, and justice-involved individuals
4. Sustainably support LF work	Sustaining LF services requires equitable pay through Medicaid, Medicare, and value-based care, supported by advocacy and accountability to ensure workforce stability
5. Support LF research priorities	Research should assess LF integration, effectiveness, workforce retention, and cost-efficiency to inform best practices and ensure sustainable, equitable implementation

LF, linkage facilitations; OUD, opioid use disorder.

### 4.1 Enhance training guides for linkage facilitators, and critically, for supervisors of linkage facilitators and other staff working at agencies with LF programs

Only recently has the position of “linkage facilitator” been acknowledged as a unique and independent role for professionals serving individuals with OUD (8, 41). The LF field would benefit from cross-cutting guidelines that could serve as best practices. However, such guidelines must be developed to not conflict with the standards of the wide swath of professional backgrounds from which linkage facilitators may originate (e.g., certified PRSS, CHW, licensed clinicians). Given the current shortage in the behavioral health workforce, standards should avoid creating unnecessary barriers for professionals to work as linkage facilitators, such as requiring higher-level certifications or licenses for LF roles. Perhaps more urgently, however, there is a need for development of trainings and resources for both supervisors and coworkers of those performing LF for people with OUD. Robust, responsive, and compassionate supervision has been identified as a critical aspect of supporting successful linkage facilitators (11, 67, 74, 77). Furthermore, research has documented challenges arising from other agency staff, including stigma, philosophical clashes, and lack of role clarity (37, 38, 61). Leadership within each organization delivering LF would be well-positioned to equip supervisors and coworkers with resources and knowledge to respect and support linkage facilitators, but development of training guides would facilitate this process much more effectively. To translate these insights into actionable strategies, we organize the discussion around five key areas where targeted investment, research, and infrastructure can advance the field of linkage facilitation for OUD.

### 4.2 Develop an accessible resource library for organizations providing LF or interested in initiating LF services

While the expansion and synthesis of trainings materials is an important initial step in supporting the growing LF workforce, dissemination of these and other LF-related resources is urgently needed. We recommend that the field invest in a centralized resource-sharing hub where organizations who offer LF for persons with OUD, or are interested in initiating LF services, can access up-to-date information and relevant tools. For example, organizations considering hiring linkage facilitators may wish to read about recent research findings, particularly service delivery models, that could aid in development of an approach tailored to their program (and which, backed by evidence, may be more compelling for leadership, stakeholders, and funding agencies). Those organizations that offer LF could benefit from access to tools for measurement of fidelity, examples of official documentation (e.g., standard operating procedures), or recommendations for career advancement that would support the LF workforce, but which may be underutilized. Guidance on how organizations can address hiring restrictions based on lived experience (e.g., history of incarceration, drug offenses) would also be helpful. Additionally, this type of central hub would allow for networking between linkage facilitators, organizations providing LF services, researchers, and others interested in LF who may hope to connect or consult with practitioners and other experts. Although initiation of this type of resource center would be an investment of time, energy, and resources, it would pay dividends towards supporting the future LF workforce—both specifically related to OUD services and more broadly.

### 4.3 Improve understanding of populations who may require uniquely tailored LF services in addition to support related to OUD

As the need for LF services for people with OUD continues to grow, and the LF workforce expands to meet it, the diversity of individuals with OUD becomes an increasingly important consideration for linkage facilitator training and education. For example, pregnant and postpartum people may require linkage to specialized medical services and assistance navigating disparate systems (e.g., child welfare, social agencies). Individuals with OUD and criminal legal system (CLS) involvement can experience difficulty accessing employment or safe/supportive housing and may face stigma, discouragement, or denial from CLS staff related to some types of OUD treatment, particularly MOUD (41). LGBTQ+ individuals may experience mistreatment within health settings, leaving them underserved and isolated from quality health care (103–105), which could be a key opportunity for linkage facilitators to engage in knowledge translation and advocacy.

Furthermore, youth and emerging adults with OUD represent a unique demographic with rising rates of overdose (106), yet LF for this population—including defining who is best-positioned to provide LF services—remains understudied. There is a distinct need for research related to LF for these and other unique populations of individuals with OUD to better understand best practices for advocacy, communication, and tailored service needs. Additionally, given that some LF models include linkage facilitators with direct lived experience (e.g., PRSS), work is direly needed to expand understanding and develop recommendations for supervisors and organizations employing linkage facilitators who themselves identify as members of these populations. This will more fully support the LF workforce to provide quality tailored services to diverse individuals with OUD who may have unique needs related to their identities and circumstances.

### 4.4 Find ways to sustainably support LF work

Sustainably supporting LF services will require payment structures that prioritize equitable compensation, as financial instability and inadequate pay contribute to high turnover in this essential workforce (19, 21). Better aligning compensation models with Medicaid, Medicare, and value-based care models presents a solution, recognizing the importance of linkage facilitators in improving patient outcomes and reducing healthcare costs. While Medicare has integrated care billing mechanisms that could support LF services, they are rarely utilized (107, 108). Medicaid varies from state to state, but growing trends in covering peer and CHW services [e.g., (109)] provide a potential route to supporting LF (77, 110). Value-based care can support linkage facilitators by aligning financial incentives with patient outcomes, ensuring that the essential work of linkage facilitators in connecting individuals with care is recognized and adequately compensated, while also improving healthcare efficiency and patient recovery outcomes through integrated care models (111, 112). Ensuring payment structures are available and equitable across the nation will likely require coordinated advocacy (107, 113). Additionally, accountability structures (e.g., the proposed central LF workforce hub in section 4.2) may need to be instituted to ensure that organizations equitably reflect the provisions of such payment structures in their linkage facilitators’ compensation. This combination of equitable pay and policy reform is essential for maintaining a stable LF workforce, thereby enhancing care continuity and promoting long-term recovery for individuals with OUD.

### 4.5 Research priorities

Studies are needed that examine the general integration of LF services for OUD across different contexts and with diverse populations. Observational research can help map how LF is implemented across various healthcare, community, and legal settings. Such research can compare existing LF models to identify best practices, challenges, and potential service delivery gaps (8, 114). Moreover, the diversity of linkage facilitator backgrounds (e.g., peers, CHWs, licensed clinicians) requires comparative research on how different types of LF professionals function within these settings and with varying patient populations.

Clinical trials aimed at identifying the effectiveness of specified OUD LF interventions would help optimize the impact of LF on both individual and system-level outcomes. Such trials should prioritize identifying the causal mechanisms of effectiveness—whether through increasing treatment engagement, reducing relapse rates, or promoting long-term recovery (114). This type of research is crucial to optimizing the impact of LF on both individual and system-level outcomes. Hybrid studies that are also focused on implementation can develop fidelity standards and identify best strategies for LF implementation, leading to more rapid research translation (115). This will also ensure consistency in delivering LF services, thereby enhancing the reliability of the research and the quality of care provided in real-world settings.

There is a need for research focused on LF workforce outcomes and the identification of factors that prevent linkage facilitator burnout and support retention. Given the high turnover rates in the behavioral health workforce, it is essential to explore the protective factors that can enhance job satisfaction, reduce stress, and promote long-term workforce stability (116). Research informed by existing occupational health theories, such as the Job Demands-Resources model (117) or the Conservation of Resources theory (118), could offer valuable frameworks for understanding how to balance the demands placed on LF workers with the resources available to them. This line of research should examine factors such as workload, role clarity, access to professional development, and support from colleagues, all of which can play a role in either mitigating or exacerbating burnout. Within this broader focus, understanding effective leadership and supervision models is particularly important. Leadership approaches that foster a positive, supportive work environment, promote autonomy, and provide trauma-informed supervision are likely to enhance job satisfaction and retention. Research that explores how different leadership and supervisory styles impact burnout, job satisfaction, and overall workforce retention will be essential to optimizing LF service delivery and ensuring a sustainable, resilient workforce (72, 119, 120).

Cost-benefit and cost-effectiveness analyses of LF models will help quantify both the direct and indirect financial impacts of these interventions. By establishing the costs associated with implementing LF, as well as the long-term savings generated through improved patient outcomes—such as reductions in hospitalizations, relapses, and criminal legal involvement—these evaluations can provide a strong case for integrating LF services into reimbursement structures (121, 122). Importantly, economic evaluations can also inform equitable pay for linkage facilitators by showing the economic value of their contributions to the healthcare system and patient recovery to inform development of fair compensation models, reducing workforce turnover and contributing to workforce sustainability.

#### 4.5.1 Strengths and limitations

This narrative review offers a timely synthesis of the fragmented literature on linkage facilitation for individuals with OUD, providing practical insights for workforce development and organizational support. A key strength lies in the interdisciplinary expertise of the authorship team, which allowed for integrative insights drawn from practice and various research methods across the field (24), behavioral health, and community-based systems. However, the review is limited by the subjective nature of narrative synthesis and the absence of a systematic search protocol, which may have introduced selection bias (123). Additionally, the current evidence base on LF and OUD practices remains nascent, with limited high-quality, empirical studies directly examining workforce experiences, intervention effectiveness, or implementation strategies. Future research should prioritize rigorous, longitudinal, and implementation-focused studies to build a more robust evidence base and inform scalable models for supporting this emerging workforce (8, 114).
